# Which tinnitus-related aspects are relevant for quality of life and depression: results from a large international multicentre sample

**DOI:** 10.1186/1477-7525-12-7

**Published:** 2014-01-14

**Authors:** Florian Zeman, Michael Koller, Berthold Langguth, Michael Landgrebe

**Affiliations:** 1 Centre for Clinical Studies, University Hospital Regensburg, Franz-Josef-Strauss-Allee 11, Regensburg 93053, Germany; 2 Department of Psychiatry and Psychotherapy, University of Regensburg, Universitätsstraße 31, Regensburg 93053, Germany; 3 Department of Psychiatry, Psychosomatics and Psychotherapy, kbo-Lech-Mangfall-Klinik Agatharied, Norbert-Kerkel-Platz, Hausham (Obb) 83734, Germany

**Keywords:** Tinnitus, Tinnitus handicap inventory, Tinnitus severity, Quality of life, Depression

## Abstract

**Background:**

The aim of the present study was to investigate, which aspects of tinnitus are most relevant for impairment of quality of life. For this purpose we analysed how responses to the Tinnitus Handicap Inventory (THI) and to the question “How much of a problem is your tinnitus at present” correlate with the different aspects of quality of life and depression.

**Methods:**

1274 patients of the Tinnitus Research Initiative database were eligible for analysis. The Tinnitus Research Initiative database is composed of eight study centres from five countries. We assessed to which extent the Tinnitus Handicap Inventory (THI) and its subscales and single items as well as the tinnitus severity correlate with Beck Depression Inventory (BDI) score and different domains of the short version of the WHO-Quality of Life questionnaire (WHO-QoL Bref) by means of simple and multiple linear regression models.

**Results:**

The THI explained considerable portions of the variance of the WHO-QoL Physical Health (R^2^ = 0.39) and Psychological Health (R^2^ = 0.40) and the BDI (R^2^ = 0.46). Furthermore, multiple linear regression models which included each THI item separately explained an additional 5% of the variance compared to the THI total score. The items *feeling confused from tinnitus*, *the trouble of falling asleep at night, the interference with job or household responsibilities*, *getting upset from tinnitus,* and *the feeling of being depressed* were those with the highest influence on quality of life and depression. The single question with regard to tinnitus severity explained 18%, 16%, and 20% of the variance of Physical Health, Psychological Health, and BDI respectively.

**Conclusions:**

In the context of a cross-sectional correlation analysis, our findings confirmed the strong and consistent relationships between self-reported tinnitus burden and both quality of life, and depression. The single question “How much of a problem is your tinnitus” reflects tinnitus-related impairment in quality of life and can thus be recommended for use in clinical routine.

## Introduction

Tinnitus is the perception of sound within the human ear in the absence of any external acoustic stimuli. With prevalence rates between 2.4% and 20.1% [1] tinnitus represents a frequent disorder. The rather large range in the reported prevalence rates can be explained by the highly variable definitions used in the different epidemiologic studies [2].

The extent to which tinnitus impairs quality of life is highly variable. Many people remain unaffected by the phantom sounds, whereas others are severely impaired and may even become suicidal [3]. Various questionnaires have been developed to assess tinnitus severity or tinnitus-related impairment. Although these questionnaires have been cross-validated with each other, only limited information exists about their relation to quality of life measurements. Nevertheless, the use of non-tinnitus-specific instruments for the assessment of quality of life has been recommended by an expert consensus [4]. Existing studies that investigated the relationship between tinnitus severity and quality of life include rather small samples and have used different measurements for tinnitus severity and impaired quality of life [5-10]. A study using the Short Form Health Survey (SF-36) for assessing patients’ quality of life showed that 43% of patients with tinnitus also had impaired quality of life or a high level of distress or both [11]. Furthermore, many tinnitus patients are known to suffer from insomnia [12], which has a considerable impact on the quality of life [13].

The aim of this study was to investigate the extent to which self-reported tinnitus burden as assessed by the Tinnitus Handicap Inventory (THI) ─ one of the most widely used tinnitus questionnaires ─ and the answer to one single question (“How much of a problem is your tinnitus”) correlate with the different aspects of quality of life and depression. For assessing quality of life, we used the WHO-QoL Bref questionnaire, which was developed for quantifying health-related quality of life, because this questionnaire has already been proven to be suitable for assessing tinnitus-related impairment [10]. In addition, we assessed depressive symptoms with the Beck Depression Inventory (BDI). Data from an international database set up by the Tinnitus Research Initiative [14] were analyzed. Eight study centres from five different countries have contributed data to this project.

The main objective of the study was to investigate correlations between tinnitus burden, quality of life and depression. In particular, we wanted to assess which specific aspects of tinnitus, measured by the 25 items of the Tinnitus Handicap Inventory, are relevant for impairment in quality of life. This information has a direct implication for the development of therapeutic interventions and for clinical tinnitus management. To our knowledge this is the first study which addresses these important questions in a large multinational sample.

## Material and methods

### Database

The data analysis was based on data of the Tinnitus Research Initiative database. The primary objective of this database is the collection of a standardized set of data from studies of various designs on patient characteristics, treatments and outcomes, for delineating different tinnitus subtypes of tinnitus and for identifying predictors for individual treatment response [14].

The Tinnitus Research Initiative database includes data from studies of various designs (randomized controlled, longitudinal, one-armed observational and cross-sectional baseline) and different countries (Germany, Belgium, Brazil, Argentina, USA and Switzerland). All patients were treated as outpatients. All studies comply with a pre-specified standardized documentation set [14]. Collection of data for the Tinnitus Research Initiative database was approved by the Ethics Committee of the University of Regensburg, Germany (reference number 08/046).

Data management was conducted according to the Data Handling Plan (TRI-DHP V05, 21.02.2011), which can be found at http://database.tinnitusresearch.org/.

The data set released on November 1st, 2012 contained n = 2542 patients, of which n = 1274 were eligible for analysis.

### Assessments

The Case Report Form (CRF) of the Tinnitus Research Initiative database contains different types of information (medical history, audiological examinations, tinnitus-related questionnaires, self-assessment instruments for depression, and quality of life) in different languages (German, Dutch, Portuguese, and Spanish) [15]; all questionnaires were collected in a standardized manner [14].

One of the most common questionnaires for assessing the tinnitus related handicap in daily life is the Tinnitus Handicap Inventory (THI) [16]. The THI is mainly used for the stratification of patients with tinnitus according to tinnitus related handicap. The THI consists of 25 items, each with the three response options *yes* (4 points), *sometimes* (2 points), and *no* (0 points), resulting in a total score range from 0 to 100. This score can be categorized into the following five categories: *slight tinnitus* (0 to 16), *mild tinnitus* (18 to 36), *moderate tinnitus* (38 to 56), *severe tinnitus* (58 to 76), and *catastrophic tinnitus* (78 to 100). The THI can be divided into three subscales: functional, emotional, and catastrophic [16]. Notably the three factor structure has been questioned, since it was solely based on content validity of the domains and was not subjected to empirical validation of the questionnaire structure [17]. We investigated the relevance of the total score, the subscale scores and each single item.

In addition, patient’s subjective perception of tinnitus severity was assessed by the single question “How much of a problem is your tinnitus at present”, for which five answer options were given, ranging from *no problem* to *a very big problem*[18]. All questionnaires analyzed for predicting quality of life and depression are summarized in Table 1.

**Table 1:** Tinnitus-related assessments

**Measure**	**Content**	**Time frame item example**
**Tinnitus Handicap Inventory (THI)**	25 items	Time frame not specified
	3 response options	
	○ yes (4 points) ○ sometimes (2 points) ○ no (0 points)	“Because of your tinnitus do you feel frustrated?”
	Summary score 0 to 100	
	A higher score denotes higher tinnitus-related handicap	
**THI functional subscale**	Score 0 to 44, reflects role limitations in the areas of mental, social, occupational and physical functioning	“Because of your tinnitus do you often feel tired?”
**THI emotional subscale**	Score 0 to 36, representing a broad range of affective responses to tinnitus	“Does your tinnitus make you angry?”
**THI catastrophic subscale**	Score 0 to 20, reflects patients’ desperation, inability to escape from tinnitus, perception of having a terrible disease, lack of control and inability to cope	“Because of your tinnitus do you feel desperate?”
**Tinnitus severity**	One question about tinnitus severity	Present
	○ no problem (1 point) ○ a small problem (2 points) ○ a moderate problem	“How much of a problem is your tinnitus at present”
	(3 points)	
	○ a big problem (4 points) ○ a very big problem (5 points)	

To measure patient’s quality of life, we used the short version of the WHO-Quality of Life questionnaire (WHO-QoL Bref) developed by the World Health Organization Quality of Life Group [19]. This questionnaire comprises 26 items, of which 24 measure the domains physical health (seven items), psychological health (six items), social relationships (three items), and environment (eight items). The domain scores are normalized and range from 4 to 20. The remaining two items measure the overall quality of life and general health. Since the present analysis focused on specific aspects of the multidimensional construct quality of life these two general items were not considered in the analyses.

For measuring depressive symptoms, we used the Beck Depression Inventory [20] consisting of 21 items with four response options resulting in a total score between 0 and 63. This score can be categorized into the four categories *minimal depression* (0 to 9), *mild depression* (10 to 18), *moderate depression* (19 to 29), and *severe depression* (30 to 63). An overview about Quality of life questionnaires and BDI is given in Table 2.

**Table 2:** Quality of life & depression

**Measure**	**Content**	**Time frame item example**
**WHO-QoL Bref domain1**	**Physical health**	Last 4 weeks
	7 items	“How well are you able to go around?”
	5 response options	
	Score 4 to 20	
	A higher score denotes higher physical health	
**WHO-QoL Bref domain2**	**Psychological health**	Last 4 weeks
	6 items	“How much do you enjoy life?”
	5 response options	
	Score 4 to 20	
	A higher score denotes higher psychological health	
**WHO-QoL Bref domain3**	**Social relationships**	Last 4 weeks
	3 items	“How satisfied are you with your personal relationships?”
	5 response options	
	Score 4 to 20	
	A higher score denotes better social relationships	
**WHO-QoL Bref domain4**	**Environment**	Last 4 weeks
	8 items	“To what extend do you have the opportunity for leisure activities?”
	5 response options	
	Score 4 to 20	
	A higher score denotes better environment	
**Beck Depression Inventory (BDI)**	**Depression**	Last 2 weeks
	21 items	“How sad do you feel?”
	4 response options	
	Score 0 to 63	
	A higher score denotes higher depression	

The order of questionnaires throughout all studies was: (1) THI, (2) TBF-12 (not considered), (3) Tinnitus Severity (single question), (4) BDI, and (5) WHO-QoL.

### Statistical analysis

Patient characteristics are summarized as median values and interquartile ranges (first to third quartiles) for continuous variables and as frequency counts and percentages for categorical data. Simple linear regression models were calculated to analyze the influence of THI total score, THI subscales, and tinnitus severity on quality of life and depression. To identify single THI items as predictors of quality of life and depression, we calculated multiple linear regression models. A p-value ≤ 0.05 was considered statistically significant. All analyses were done with IBM SPSS Statistics 20.0 and SAS 9.3 (SAS Institute Inc., Cary, NC, USA) and were conducted according to the Standard Operating Procedure (TRI-SA V01, 09.05.2011), thereby following a study-specific Statistical Analysis Plan (SAP-010) that was written according to the SAP template (TRI-SAP 006, 12.05.2011) (see http://database.tinnitusresearch.org/).

## Results

### Patient characteristics

For 1274 out of 2542 patients, full datasets including all domains of the WHO-QoL Bref, the BDI and either the THI (1260 datasets, 99%) or tinnitus severity (1165 datasets, 91%) were available. These data were included in this analysis. The median age was 52 years (IQR: 43 to 61 years), and the median tinnitus duration was 5.0 years (IQR: 1.6 to 11.9 years). Further patient and tinnitus-related baseline characteristics are summarized in Table 3. The patient sample with full datasets, which was included in the analysis, did not differ from the non-included patients with respect to gender (male 65.9% vs. 65.4%), age (mean age 52.0 vs. 52.1 years), tinnitus duration (mean tinnitus duration 100.6 vs. 95.9 months) and THI score (mean THI score 47.7 vs. 47.3).

**Table 3:** Patient characteristics (n = 1274)

	
**Age (years)**, median (IQR)	52 (43; 61)
**Tinnitus duration (years)**, median (IQR)	5.0 (1.6; 11.9)
**Sex** (N,%)	
Men	839 (66%)
Women	435 (34%)
**THI totalscore,** median (IQR)	46 (30; 66)
**Tinnitus severity,** (N,%*)	
Not a problem	16 (1%)
A small problem	139 (11%)
A moderate problem	459 (36%)
A big problem	418 (33%)
A very big problem	133 (10%)
**WHO-QoL Bref: physical health,** median (IQR)	14.9 (12.6; 16.6)
**WHO-QoL Bref: psychological health,** median (IQR)	14.4 (12.0; 16.0)
**WHO-QoL Bref: social relationships,** median (IQR)	14.7 (12.0; 16.0)
**WHO-QoL Bref: environment** median (IQR)	16.0 (14.5; 17.5)
**BDI total score,** median (IQR)	9 (5; 16)

*IQR*: interquartile range.

%^*^: do not add up to 100% because of occasional missing values.

### THI summary score and self-reported severity

**Figure 1:**
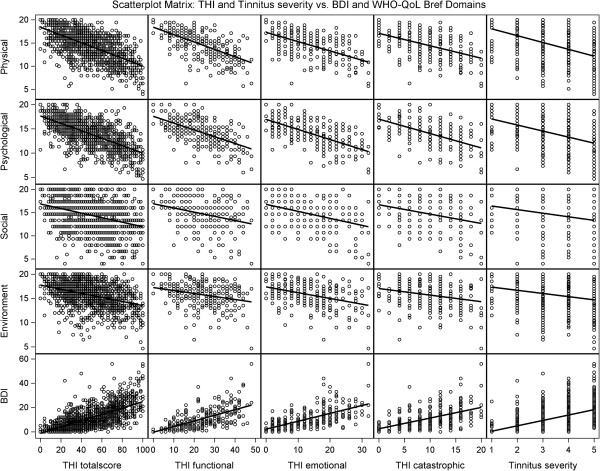
Scatterplot matrix: THI, THI subscales, and Tinnitus severity vs. WHOQOL Bref domains and BDI.

Table 4 shows the results of the simple linear regressions of THI total score, THI subscales (functional, emotional, and catastrophic), and self-reported tinnitus severity on the four WHO-QoL-Bref domains and the BDI summary score. The highest variance explanation was found between the total scores of THI and BDI with an R^2^ value of 0.46. The R^2^-values for WHO-QoL Bref Domain 1 (physical health) and 2 (psychological health) were higher (R^2^_Dom1_ = 0.39, R^2^_Dom2_ = 0.40) than those for Domain 3 (social relationships) and Domain 4 (environment) (R^2^_Dom3_ = 0.13, R^2^_Dom4_ = 0.16). Furthermore, the THI subscales explained less variance than the THI total score. Still tinnitus severity, consisting of one question (scale from 1 to 5), explained 18% of the variance of the WHO-QoL Bref subscale physical health and 16% of the variance of the subscale psychological health. The highest coefficient of determination was again found by explaining the BDI (R^2^ = 0.20). Linear relationships are shown as a scatterplot matrix in Figure 1. The slope of the regression line is given by the regression coefficient B (Table 4). For example, the linear regression of THI on physical health with a B of −0.08 represents a reduction of one point in physical health for an approximately 12 points increase in the THI total score.

**Table 4:** Simple linear regressions --- THI total score (n = 1260), THI subscales (n = 1260), and Tinnitus severity (n = 1165) on WHO-QoL Bref domains and BDI

	**Physical health**		**Psychological health**		**Social relationship**		**Environment**		**BDI**	
	**R**^**2**^	**B, p-value**	**R**^**2**^	**B, p-value**	**R**^**2**^	**B, p-value**	**R**^**2**^	**B, p-value**	**R**^**2**^	**B, p-value**
**THI total**	0.39	−0.08, **<0.001**	0.40	−0.08, **<0.001**	0.13	−0.05, **<0.001**	0.16	−0.04, **<0.001**	0.46	0.26, **<0.001**
**THI functional**	0.37	−0.16, **<0.001**	0.34	−0.14, **<0.001**	0.11	−0.09 **<0.001**	0.08	−0.06, **<0.001**	0.36	0.47, **<0.001**
**THI emotional**	0.34	−0.20, **<0.001**	0.41	−0.21, **<0.001**	0.17	−0.15, **<0.001**	0.16	−0.12, **<0.001**	0.39	0.65, **<0.001**
**THI catastrophic**	0.21	−0.27, **<0.001**	0.30	−0.30, **<0.001**	0.11	−0.20, **<0.001**	0.07	−0.14, **<0.001**	0.26	0.88, **<0.001**
**Tinnitus severity**	0.18	−1.46, **<0.001**	0.16	−1.25, **<0.001**	0.05	−0.78, **<0.001**	0.06	−0.66, **<0.001**	0.20	4.44, **<0.001**

R^2^_,_ coefficient of determination; B, regression coefficient.

**Figure 2:**
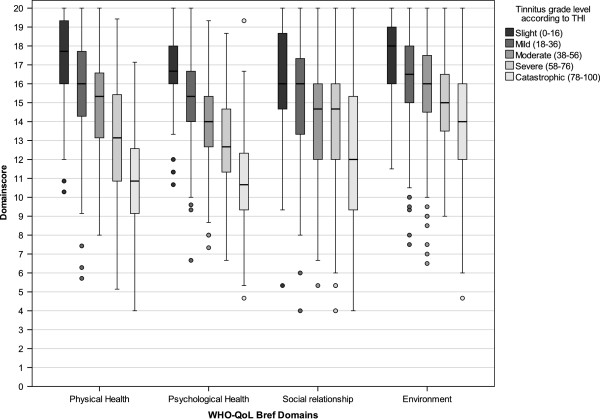
Boxplots of WHO-QoL Bref domain scores categorized according to THI tinnitus grade level.

**Figure 3:**
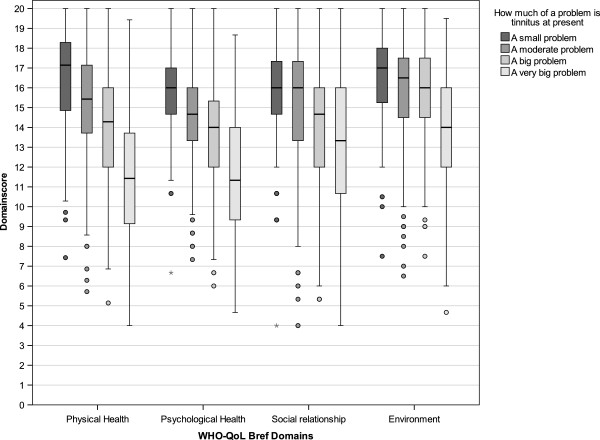
Boxplots of WHO-QoL Bref domain scores categorized according to self-reported tinnitus severity.

**Figure 4:**
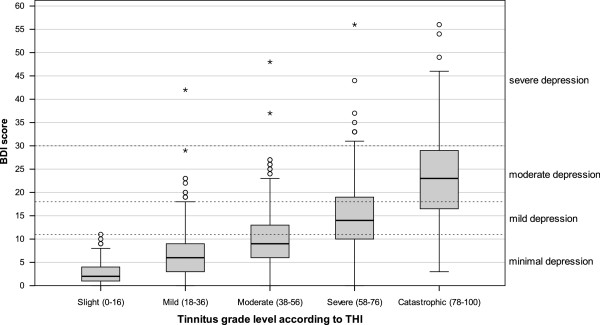
Boxplots of BDI scores categorized according to THI tinnitus grade level (dotted lines show BDI categories).

**Figure 5:**
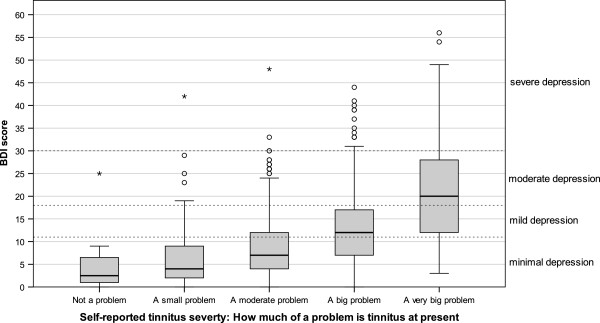
Boxplots of BDI scores categorized according to self-reported tinnitus severity (dotted lines show BDI categories).

To visualize the correlations of THI categories and self-reported tinnitus severity with the WHO-QoL Bref Domains, boxplots are shown in Figures 2 and 3, whereas the relationships to BDI are shown in Figures 4 and 5.

### THI items

By adding all THI items to a multiple regression model for each WHO-QoL Bref domain as well as for BDI, 15 out of 25 items (questions 4, 5, 6, 7, 11, 12, 13, 15, 16, 20, 21, 22, 23, 24, and 25) remained significant in one of the regression models (Table 5). Compared to the THI-total score models, the R^2^-values for each model were considerably higher (+0.02 to +0.09). Items with the highest influence on quality of life and depression regarding the regression coefficient B were “*feeling confused from tinnitus”*, “*the trouble of falling asleep at night”,* “*the interference with job or household responsibilities”*, “*getting upset from tinnitus”,* and “*the feeling of being depressed”*. By reducing all models to the significant variables, the R^2^-values remained almost the same and varied between 0.16 and 0.49 (data not shown). These values were still higher than the total score models of THI.

**Table 5:** Multiple linear regressions of all 25 THI items on WHO-QoL Bref Domains and BDI (n = 1227)

THI No*	**THI items**	**Physical health**		**Psychological health**		**Social relationships**		**Environment**		**BDI**	
		**B**	**p**	**B**	**p**	**B**	**p**	**B**	**p**	**B**	**p**
	(Constant)	17.79	0.00	17.02	0.00	16.33	0.00	16.93	0.00	1.47	0.02
1F	Because of your Tinnitus is it difficult for you to concentrate?	0.04	0.79	0.01	0.92	0.08	0.65	0.18	0.18	0.18	0.65
2F	Does the loudness of your Tinnitus make it difficult for you to hear people?	−0.08	0.43	0.07	0.42	0.06	0.62	0.03	0.72	−0.02	0.95
3E	Does your Tinnitus make you angry?	0.09	0.48	0.06	0.58	0.14	0.37	−0.05	0.69	−0.05	0.89
4F	Does your Tinnitus make you confused?	**−0.42**	**0.00**	**−0.40**	**0.00**	**−0.38**	**0.02**	**−0.52**	**0.00**	**1.60**	**0.00**
5C	Because of your Tinnitus are you desperate?	−0.22	0.12	**−0.41**	**0.00**	0.01	0.93	0.22	0.10	**1.00**	**0.01**
6E	Do you complain a great deal about your Tinnitus?	−0.08	0.44	0.18	0.07	**0.34**	**0.02**	−0.11	0.27	−0.41	0.17
7F	Because of your tinnitus do you have trouble falling asleep at night?	**−0.73**	**0.00**	−0.09	0.33	0.03	0.83	**−0.19**	**0.03**	**0.89**	**0.00**
8C	Do you feel as though you cannot escape from your Tinnitus?	0.07	0.59	−0.10	0.34	0.06	0.68	−0.10	0.36	0.26	0.44
9F	Does your Tinnitus interfere with your ability to enjoy social activities?	−0.18	0.14	0.04	0.72	−0.01	0.92	0.00	1.00	−0.03	0.93
10E	Because of your Tinnitus do you feel frustrated?	0.08	0.55	−0.04	0.78	−0.15	0.41	0.20	0.12	0.13	0.73
11C	Because of your Tinnitus do you feel that you have a terrible disease?	−0.06	0.61	0.16	0.12	0.00	0.99	**−0.21**	**0.04**	0.51	0.10
12F	Does your Tinnitus make it difficult to enjoy life?	0.23	0.09	−0.13	0.29	0.00	1.00	**0.35**	**0.01**	0.02	0.97
13F	Does your Tinnitus interfere with your job or household responsibilities?	**−0.76**	**0.00**	**−0.42**	**0.00**	−0.11	0.53	**−0.53**	**0.00**	**1.54**	**0.00**
14F	Because of your Tinnitus do you find that you are often irritable?	−0.09	0.46	−0.19	0.10	−0.09	0.55	0.04	0.70	0.35	0.31
15F	Because of your Tinnitus is it difficult for you to read?	−0.18	0.10	**−0.21**	**0.03**	**−0.30**	**0.03**	−0.03	0.78	0.27	0.37
16E	Does your Tinnitus make you upset?	**−0.29**	**0.03**	**−0.25**	**0.04**	**−0.53**	**0.00**	**−0.78**	**0.00**	**0.89**	**0.02**
17E	Do you feel that your Tinnitus has placed stress on your relationships with members of your family and friends?	0.09	0.49	0.06	0.60	−0.26	0.11	0.20	0.10	0.44	0.22
18F	Do you find it difficult to focus your attention away from your Tinnitus and on to other things?	−0.21	0.11	−0.21	0.09	0.05	0.76	0.02	0.88	0.05	0.88
19C	Do you feel that you have no control over your Tinnitus?	0.08	0.47	−0.02	0.83	−0.17	0.23	0.16	0.12	−0.29	0.36
20F	Because of your Tinnitus do you often feel tired?	**−0.47**	**0.00**	−0.15	0.13	0.11	0.43	−0.09	0.37	**0.82**	**0.01**
21E	Because of your Tinnitus do you feel depressed?	−0.24	0.10	**−0.60**	**0.00**	**−0.70**	**0.00**	−0.20	0.12	**1.78**	**0.00**
22E	Does your Tinnitus make you feel anxious?	**−0.35**	**0.00**	**−0.28**	**0.01**	**−0.30**	**0.05**	**−0.23**	**0.04**	**0.64**	**0.05**
23C	Do you feel you can no longer cope with your Tinnitus?	−0.13	0.32	**−0.31**	**0.01**	−0.11	0.53	−0.21	0.08	0.13	0.72
24F	Does your Tinnitus get worse when you are under stress?	**−0.19**	**0.04**	−0.06	0.43	−0.05	0.64	−0.04	0.66	0.31	0.20
25E	Does your Tinnitus make you feel insecure?	−0.08	0.47	**−0.21**	**0.05**	0.03	0.86	−0.16	0.14	**1.00**	**0.00**
	R^2^ (adjusted R^2^)	0.45 (0.44)		0.45 (0.44)		0.17 (0.15)		0.26 (0.25)		0.50 (0.49)	

Each column represents a multiple linear regression model with one of the WHO-QoL Bref Domains or the BDI as the dependent variable and all 25 THI items as independent variables. *letters represent THI subscales; F: functional, E: emotional, C: catastrophic; R^2^ (adjusted R^2^), coefficient of determination (coefficient of determination adjusted for the number of exploratory items); B, regression coefficient, p, p-value; all significant items (p < 0.05) are printed in boldface.

## Discussion

In this large international sample the THI and all its subscales but also the simple tinnitus severity question “How much of a problem is your tinnitus” strongly predicted tinnitus related impairment in quality of life. The THI score was particularly related to “depressive symptoms”, “physical health”, and “psychological health”, whereas its relation to “social relationship” and “environment” was less pronounced.

The strong relation between THI and BDI confirmed earlier studies [21,22] and was to be expected, since the THI was validated against the BDI [16]. The finding that patients with severe (THI between 58 and 76) and catastrophic tinnitus (THI ≥78) showed substantial depressive symptoms (Figure 4) is clinically highly relevant and underscores the usefulness of the THI as a screening instrument for co-morbid depression [23,24]. This has the implication that high scores in the THI (and probably also in other tinnitus questionnaires assessing tinnitus burden like the TQ or THQ) should be an indicator for the clinician to further evaluate potential psychiatric comorbidities. The same is true for the question “How much of a problem is your tinnitus?”; patients answering “a big problem” or “a very big problem” clearly showed higher BDI scores (Figure 3). The substantial correlations between the answer to “How much of a problem is your tinnitus?” and WHO-QoL scores indicate that one simple question that every tinnitus patient can be easily asked in a clinical context provides a good orientation for tinnitus-related impairment of quality of life.

More recently, the high correlation between the THI and the BDI has been argued to be mainly due to a significant content overlap [21]. However, our results of high correlations between the THI and QoL scores indicate that high THI scores reflect a significant impairment in quality of life, independent of a potential content overlap of single items between the THI and the BDI [25]. Notably, the observed high correlation between the THI and the BDI does not provide any information on the direction of the effect [26]. Tinnitus may cause depressive symptoms, and depressive symptoms may contribute to tinnitus impairment; both symptoms could result from third factors, for example, personal characteristics [27,28].

Showing an explanation of 30% to 45% of the variance in the quality of life scores, our data confirm the validity of the THI for assessing tinnitus-related impairment in quality of life, thus proving the relevance of tinnitus for the quality of life. Our data further indicate that tinnitus strongly influence physical and psychological health and, to a lesser extent, social relationships and environment.

The investigation of the three factors of the THI showed that both the functional and the emotional factor had a relatively strong influence on QoL scores, whereas the catastrophic factor was less relevant. However, none of the subscales was clearly superior to the total THI score in explaining the quality of life sub-scores. This finding questions the usefulness of the different factors and suggests that the THI should be rather considered as a one-factor instrument, in line with earlier investigations which questioned the factor structure of the THI [17].

The analysis of the answers to the individual items of the THI showed that 15 out of the 25 items of the THI are significantly related to the BDI and WHO-QoL scores. These 15 items explain more variance of BDI and WHO-QoL scores than the THI total score (Table 5). Compared to a recently published short version of the THI, named THI-12 [29], only 4 of the 15 items are included in the THI-12. The other 8 items of the THI-12 are items without any significant explanatory value on quality of life. Out of the 5 THI items with the highest explanatory value on WHO-QoL scores (4,7,13,16,21), only one item (interfering with job or household responsibilities) is also part of the THI-12 version. This fact shows, that the 12 selected items for the THI-12 mainly explain tinnitus burden and do not consider quality of life. Thus our analysis has identified a subset of potentially suitable items for a short version of the THI reflecting quality of life in tinnitus patients and not tinnitus severity itself. Nevertheless, one should carefully consider, whether a further short tinnitus scale is needed (e.g. [18]). First, our findings have to be confirmed in independent samples and in longitudinal studies. Second suboptimal psychometric properties of certain items have to be traded off against the total amount of data available from a given standardized instrument and the comparability of data across studies. Our data support item-specific analysis of the THI, especially if specific aspects of tinnitus are the focus of research.

The items with the highest influence on quality of life and depression were “*feeling confused from tinnitus”*, “*the trouble of falling asleep at night”,* “*the interference with job or household responsibilities”*, “*getting upset from tinnitus”,* and “*the feeling of being depressed”*. Sleeping difficulties [12] and depression [26] are well known to be relevant factors influencing quality of life of tinnitus patients. Interference *with job- or household responsibilities* is highly plausible and has been proposed as a criterion for the clinical classification of tinnitus severity [30]. *Feeling confused* and *getting upset* from tinnitus are also comprehensible as relevant criteria, but their high relevance on quality of life is still somewhat surprising. Related to this finding is the earlier observation that high tinnitus severity is related to low scores in “agreeableness”, which in turn might represent a risk factor for getting upset [28]. Other aspects that could be assumed to be at least of similar relevance, such as *concentration difficulties, hearing difficulties, lack of control and escape, interference with social relationships and activities, irritability, frustration* and *angriness* did not turn out to be relevant for the QoL and depression scores.

Our findings are highly informative because of the multicenter approach and the large sample size including patients with varying degrees of tinnitus from five countries. Nevertheless, we are aware that the generalizability of the findings would profit from further studies using other data base. Different instruments for assessing tinnitus severity have recently been shown to differently reflect emotional distress of tinnitus sufferers [24].

An apparent limitation is the cross-sectional approach of our study. The obtained correlations do not allow any conclusions about the direction of effects or potential causal relationships. In order to identify which changes in tinnitus related symptoms are particularly relevant for an improvement in quality of life, longitudinal studies should be performed. A further point of concern is the fact that the single tinnitus severity question “How much of a problem is your tinnitus at present” has been asked in the context of a comprehensive tinnitus assessment, namely after the THI and the Tinnitus Impairment Questionnaire (TBF-12) had been filled in. Research showed that answers are determined to some degree by the order of the questionnaires [31-33]. It is, therefore, an interesting research question whether the predictive power of the Tinnitus Severity would be affected if the order of this single question would change (e.g., either first or last in the measurement battery).

Nevertheless, our results point to implications for the clinical management of tinnitus. First, our findings validate the single question “How much of a problem is your tinnitus” as useful for obtaining a rough idea about tinnitus-related impairment in quality of life. Such a rough estimation can be useful in specific contexts, even if a more comprehensive assessment by multiple questions in validated questionnaires will remain the preferable option in most situations. Second, our results confirm the association between tinnitus and co-morbid depression in tinnitus patients, and, third, the identification of those tinnitus aspects that are highly relevant for quality of life also provides an orientation for the further development of psychotherapeutic and psychopharmacological strategies.

## Competing interests

The authors declare that they have no competing interests.

## Authors’ contributions

FZ and MK conceptualized the project and developed the statistical analysis plan (SAP). The SAP was reviewed by all authors. BL, ML and the members of the TRI database study contributed to data collection. FZ analyzed the data and drafted the manuscript. All authors contributed to the interpretation of data and to the final version of the manuscript. All authors read and approved the final manuscript.

## Tinnitus Research Initiative database study group

Ricardo Figueiredo, Andreia Aazevedo, Marcello Rates, Carolina Binetti, Ana Belen Elgoyhen, Dirk De Ridder, Sven Vanneste, Susanne Staudinger, Elmar Frank, Peter Kreuzer, Timm Poeppl, Astrid Lehner, Martin Schecklmann.
